# Transient Excursions to Membrane Core as Determinants of Influenza Virus Fusion Peptide Activity

**DOI:** 10.3390/ijms22105301

**Published:** 2021-05-18

**Authors:** Remigiusz Worch, Anita Dudek, Paulina Borkowska, Piotr Setny

**Affiliations:** 1 Institute of Physics, Polish Academy of Sciences, Aleja Lotnikow 32/46, 02-668 Warsaw, Poland; remiwo@ifpan.edu.pl (R.W.); borkowska@ifpan.edu.pl (P.B.); 2 Centre of New Technologies, University of Warsaw, Banacha 2c, 02-097 Warsaw, Poland; a.dudek@cent.uw.edu.pl

**Keywords:** influenza virus fusion peptides, peptide-membrane interactions, membrane fusion

## Abstract

Fusion of viral and host cell membranes is a critical step in the life cycle of enveloped viruses. In the case of influenza virus, it is mediated by subunit 2 of hemagglutinin (HA) glycoprotein whose N-terminal fragments insert into the target membrane and initiate lipid exchange. These isolated fragments, known as fusion peptides (HAfp), already possess own fusogenic activity towards liposomes. Although they have long been studied with the hope to uncover the details of HA-mediated fusion, their actual mechanism of action remains elusive. Here, we use extensive molecular dynamics simulations combined with experimental studies of three HAfp variants to fully characterize their free energy landscape and interaction with lipid bilayer. In addition to customary assumed peptides localization at lipid–water interface, we characterize membrane-spanning configurations, which turn out to be metastable for active HAfps and unstable for the fusion inactive W14A mutant. We show that, while the degree of membrane perturbation by surface peptide configurations is relatively low and does not show any mutation-related differences, the effect of deeply inserted configurations is significant and correlates with insertion depth of the N-terminal amino group which is the highest for the wild type HAfp. Finally, we demonstrate the feasibility of spontaneous peptide transition to intramembrane location and the critical role of strictly conserved tryptofan residue 14 in this process.

## 1. Introduction

Influenza virus entry to the cells requires fusion of viral and cellular membranes. It is mediated by a homotrimeric viral protein hemagglutinin (HA), whose HA1 subunits bind to cellular receptors and HA2 subunits are responsible for membrane remodeling [1,2]. A critical, yet still not exactly understood role in this latter respect is played by amphipatic N-terminal HA2 fragments, so-called fusion peptides (HAfp), which are directly inserted into target lipid bilayer and initiate the fusion process [3]. Their high sequence conservation and the existence of nonfunctional mutants, which nevertheless were demonstrated to embed within lipid bilayers, indicate that their role is not merely limited to act as inert anchors, but must also involve specific interplay with bilayer structure [4,5]. Synthetic peptides corresponding to only the first 20 N-terminal amino acids of HA2 have long been known to initiate the fusion of lipid vesicles and content mixing in vitro [6,7], without the mechanical action provided by the rest of the protein. Since the effect of fusion-abolishing mutations within HAfp is generally reproduced by the loss of function in complete fusion proteins [5], they have been extensively used as model systems to study basic aspects of viral fusion [8].

Experimental data concerning synthetic HAfp indicate that, whereas disordered in aqueous solution, they adopt mostly helical forms once bound to lipid structures such as membranes or micelles [9]. The N-terminal, strictly conserved part (residues 1–11) folds into a stable α-helix, followed by a kink region (residues 12–14) and a second, malleable C-terminal α-helix that starts from residue 15. Early studies, focussed on possibly shortest fusiogenic peptides composed of only 20 residues (HAfp${}_{1-20}$), suggested that both helices form a boomerang like structure, with the N-terminal arm partially inserted into hydrophobic lipid core, followed by a solvent-exposed kink, and the C-terminal segment located at lipid–water interface [10]. This view has been challenged by subsequent, nuclear magnetic resonance (NMR)-based investigation of a longer and more active 23 amino acid peptide (HAfp${}_{1-23}$), which was shown to adopt a tight helical hairpin structure in micelles, supposedly remaining at the lipid–water interface [11]. The hairpin structure is apparently not affected by further sequence elongation. According to NMR data, residues 24–28 form solvent exposed random coil [12], which suggests that they belong to a flexible tether that links the fusion peptide to the rigid coiled coil stem in a fusion ready, complete HA [13].

A sharply bent hairpin seems to be the actual fusion-active form of HAfp. Indeed, this explains well a strict conservation of several glycine residues aligned in a ridge that enables tight packing of helical arms as well as the fact that many amino acid substitutions that are known to abrogate fusion activity [8] are sterically inconsistent with the hairpin structure [11]. The activity of boomerang-like HAfp${}_{1-20}$ has been attributed to the fact that it actually exists in an ensemble of states with residual (∼11%) closed hairpin population [12], thus also explaining its lower activity compared to HAfp${}_{1-23}$. Intriguingly, however, fusion-inactive W14A mutant, with complete helices and no steric hindrance that would preclude the hairpin formation, appears to exist as an overly flexible boomerang structure [14]. It indicates that hairpin stabilization may depend not only on intra-peptide contacts but also on its interaction with environment, since the W14 side chain, apparently needed to maintain the hairpin conformation, is exposed to solvent and does not participate in interhelical interactions.

Unambiguous interpretation of the above observations is hampered by the lack of clear evidence concerning the actual peptide placement within lipid bilayer and its impact on membrane structure. The length of the N-terminal helix (11 residues) is much shorter compared to typical transmembrane helical protein domains (around 20 residues [15]). Given this notion, several NMR-based studies conclude that fusion peptides orient parallel to membrane surface and stay at the lipid–water interface [11,16]. On the other hand, measurements based on attenuated total reflection Fourier-transform infrared spectroscopy (ATR-FTIR) or spin-label electron paramagnetic resonance (EPR) suggest that peptides insert obliquely into the external membrane leaflet with the N-helix tilted on average ∼50 degrees with respect to bilayer normal [9,10,17,18], which results in buried N-terminal group and solvent-facing kink region. Moreover, recent molecular dynamics (MD) simulations indicated the possibility of fully transmembrane peptide placement which can be achieved owing to local membrane thinning caused by aqueous solvent attraction towards hydrophilic residues on both hairpin poles [19,20,21,22].

Such mixed results regarding HAfp structure and location within membrane entail the lack of consensus concerning its actual fusogenic mechanism. In this respect, multiple modes of action have been proposed, such as the promotion of lipid tails protrusions [23], lipid heads intrusions [24], membrane thinning [25,26], stabilization of positive membrane curvature [27], stabilization of negative membrane curvature [16], local membrane dehydration [28], or membrane rupture by transmembrane HAfp bundle with subsequent formation of π shaped intermediates [29]. Of the above, the likelihood of lipid tails protrusions [30] has been demonstrated via MD simulations as a viable predictor of membrane fusogenic propensity, and the extent to which it was increased by various peptide mutants was shown to correlate with their experimental activity [23,24]. In a recent simulation study of complete HA-mediated fusion [31], a particularly important role in inducing lipid tails protrusions was demonstrated for peptide configurations in which the charged HAfp N-terminus was buried within membrane core in agreement with oblique insertion mode suggested by spectroscopic measurements.

In this study, to thoroughly characterize HAfp${}_{1-23}$ configuration landscape within POPC membranes and to assess the determinants of its fusogenic activity, we combine MD simulations with spectroscopic methods. To this end, we consider wild type (wt) peptide and two mutants, E11A and W14A, that are known to have reduced or none activity, respectively [14]. We demonstrate that the peptides are rather dynamic in membrane environment. While they preferentially remain on bilayer surface, as previously indicated, it seems that the key to explain their function is to assume transient excursions into the membrane core. According to our analysis, in contrast to generally similar characteristics of surface conformations among the considered mutants, the ability to adopt deep configuration is affected by amino acid substitutions and significantly contributes to explaining the observed, mutation-related differences in activity.

## 2. Materials and Methods

### 2.1. Computational Methods

#### 2.1.1. Simulated Systems

We considered a 23-residue long HAfps: wt GLFGAIAGFIEGGWQGMVDGWYG and E11A and and W14A mutants. The N-terminus was modeled as a charged amino group, and the C-terminus was amidated. For most cases, we simulated E11 in protonated (neutral) state; however, a peptide with its charged version, denoted as wt${}^{-}$, was considered as well for membrane-spanning configurations. Simulated systems included one peptide, 162 POPC molecules (81 per leaflet) and 9337 TIP3P [32] water molecules together with sodium and chloride ions necessary to construct a neutral system consisting of a membrane slab with ∼20 Å of 0.15 mol/L NaCl solution margins on both sides. Peptides and lipids were modeled with Amber99SB-ILDNP* [33] and Amber Lipid14 [34] force fields, respectively. In addition, we considered wt HAfp simulations in transmembrane hairpin configuration using Charmm36 force field [35]. Starting geometries for surface bound and transmembrane peptides were taken from our previous runs [20]. Necessary mutations were introduced with Discovery Studio Visualiser (Biovia).

#### 2.1.2. MD Simulations

MD simulations were carried out with Gromacs software [36]. They were constructed using periodic boundary conditions, with a time step of 1 fs (2 fs for CHARMM simulations), interatomic bonds constrained using LINCS algorithm [37], van der Waals interactions smoothly shifted to 0 at 10 Å  (or cut off at 12 Å, with force-switch for CHARMM simulations), and electrostatic interactions calculated using particle mesh Ewald method [38] with 1.2 Å mesh spacing. Desired temperature and pressure of 1 bar were maintained by velocity-rescale thermostat and seimiisotropic ParinelloRahman barostat [39], respectively. Temperature replica exchange runs (tREMD) [40] were conducted using 24 or 40 replicas, at temperatures, *T*, ranging from 310 to 350 or 377 K, respectively (detailed list in the Supplementary Materials, Table S2). Exchanges were attempted every 1 ps. The diffusion of trajectories in temperature space was monitored to assure that each trajectory was able to reversibly sample the entire temperature spectrum. The summary of conducted runs is given in the Supplementary Materials (Table S3).

#### 2.1.3. Kinetic Analysis

Opening and closing of HAfp structures was considered as a two state process. The assignment of peptide configurations visited during tREMD simulations to hairpin or boomerang states was based on root mean square deviation (RMSD) of backbone heavy atoms with respect to NMR hairpin structure (pdb 2kxa, model 1) [11], with dividing threshold of 2.5 Å that corresponded to a minimum in bimodal RMSD distributions obtained at T=310 K. A set of temperature dependent kinetic equations was fitted to time evolution of open state fraction in tREMD trajectories and extrapolated to infinite time to give an estimate of equilibrium populations at T=310 K, according to method introduced by van der Spoel and Seibert [41]. All calculations were carried out with the use of g_kinetics Gromacs module. Final fractions of hairpin structures were evaluated as an average of two asymptotic infinite time estimates based on two tREMD runs that started from fully closed or fully opened conformations.

#### 2.1.4. Free Energy Calculations

Potentials of mean force for peptide translocation along the axis perpendicular to membrane plane (*z* axis) were obtained using umbrella sampling simulations based on tREMD runs with 24 replicas. The peptides were restrained to hairpin geometry (reference NMR structure, pdb 2kxa, model 1) with harmonic potential, $U_{h}$, acting on pairs of Cα atoms that were closer than 7 Å in the reference structure, with a force constant of 2.39 kcal/mol/Å${}^{2}$. Umbrella sampling was performed using biasing harmonic potentials with a force constant of 2.39 kcal/mol/Å${}^{2}$ that acted in the *z* direction between the center of mass of peptide 1–20 Cα atoms (PCOM) and the membrane center (MCOM) defined based on the positions of three terminal carbon atoms of each lipid acyl chain, located within a cylinder of 30 Å radius and *z* axis passing through PCOM. PCOM relative positions, $z_{p}=z_{PCOM}-z_{MCOM}$, were gathered for the replica run at T = 310 K. Window spacing along the reaction coordinate was 1 Å for z∈[−5,16] Å range and 2 Å intervals for z∈[18,34] Å.

To assess necessary equilibration length and the final time of production phase, $t_{end}$, for each umbrella window, we determined the time, $t_{OK}$, at which $z_{p}$ distributions gathered for $t\in [t_{OK},\frac{1}{2}\left(t_{end}+t_{OK}\right)]$ and for $t\in [\frac{1}{2}\left(t_{end}+t_{OK}\right),t_{end}]$ were similar according to Kolomogorov–Smirnov test with p>0.1. Then, $z_{p}$ distributions gathered for $t\in [t_{OK},t_{end}]$, with the requirement that $t_{end}-t_{OK}>15$ ns, were analyzed with weighted histogram analysis method [42] as implemented in Gromacs WHAM module, with a standard bootstraping error analysis.

The free energy cost of hairpin restraining with harmonic potential in membrane environment, $U_{h}$, was evaluated based on unrestrained runs using free energy perturbation formula [43]: $\Delta G_{0\to h}=-k_{B}T{\langle \exp\left(-\beta U_{h}\right)\rangle }_{\left\{F\right\}}$, where $k_{B}$ is Boltzmann constant, T=310 K. As a source of simulation frames, we used the last 500 ns of two tREMD runs (250 ns for E11A) that were started from closed and open structures (see above). A set {F} of 10,000 frames was randomly drawn from this pool such that to fulfil the proportion of open and closed structures as determined based on the kinetic analysis. The procedure was repeated 1000 times to obtain an average $\Delta G_{0\to h}$ and its error as a standard deviation. Analogous process in aqueous environment was split into simulation parts, in which the force constant of the restraining potential was gradually increased in steps 0, 0.001, 0.01, 0.1, and 1.0 to its full value used in $U_{h}$.

Free energy of unrestrained peptides as a function of PCOM position along the *z* axis, $z_{p}$, defined as above was evaluated based on the probability distributions of $p(z_{p})$ gathered during unrestrained tREMD simulations, according to $G\left(z_{p}\right)=-k_{B}T\ln\left(p(z_{p})\right)+G_{0}$ relation where $G_{0}$ is an arbitrary constant. In the case of transmembrane configurations, an additional biasing potential was introduced: $U_{b}\left(z_{p}\right)=\frac{1}{2}k{\left(z_{p}-z_{0}\right)}^{2}$ for $z_{p}>z_{0}$ and 0 otherwise, that prevented peptide from surfacing, with $z_{0}=6$ Å for wt and E11A and $z_{0}=4.5$ Å for W14A peptides. The resulting biased probability distribution, $p^{\prime }\left(z_{p}\right)$, was subsequently reweighted to obtain the unbiased $p(z_{p})$, according to the following formula: $p\left(z_{p}\right)=p^{\prime }\left(z_{p}\right)\exp\left(\beta U_{b}\left(z_{p}\right)\right)c$, with *c* being a normalization constant [44].

#### 2.1.5. Tryptophan Fluorescence Calculations

To estimate depth-dependent fluorescence quenching by brominated lipids based on our simulations, we adopted the model proposed by A. Ladokhin [45]. For a given average depth of lipid carbon-bound Br probe, $h_{m}$, and its dispersion $\sigma_{m}$ (both established based on pure POPC tREMD runs, see Table S1 for values), we calculated simulation averages of depth-dependent fluorescence profiles:

(1) $$\frac{F_{m}}{F_{0}}=\sum_{W\in \{14,21\}}{\langle \exp\left(-G\left(h_{W}-h_{m},\sigma_{m},S\right)-G\left(h_{W}+h_{m},\sigma_{m},S\right)\right)\rangle }_{MD}$$

where $\frac{F_{m}}{F_{0}}$ is the ratio of tryptophan fluorescence quenching by lipids specifically brominated at position *m*, to fluorescence without the quencher, $h_{W}$ is the position of tryptophan indole ring center along the *z* axis in simulation frames, and the two Gaussian terms, $G\left(\Delta h,\sigma ,S\right)=\frac{S}{\sigma \sqrt{2\pi }}\exp\left(\frac{{\left(\Delta h\right)}^{2}}{\sigma^{2}}\right)$, describe contributions from both membrane leaflets with *S* being the assumed quenching intensity [45] (see the Supplementary Materials, Figure S7). Given sets of $F_{m}/F_{0}$ for Br probes at (4,5),(6,7),(9,10), or (11,12) lipid acyl carbon atoms experimentally determined for each peptide, we used our simulation data to check what configuration of the respective peptide provided for the lowest root mean square error (RMSE) with respect to these values. To this end, we evaluated Equation (1) for the corresponding set of $h_{m}$ and $\sigma_{m}$ values based on simulation frames representing the considered peptide configuration, and determined RMSE subject to *S* minimization. To assess the fraction *f* and 1−f of transmembrane (TM) and surface (SURF) configurations, respectively, we assumed $F_{m}/F_{0}=f{\left(F_{m}/F_{0}\right)}_{TM}+\left(1-f\right){\left(F_{m}/F_{0}\right)}_{SURF}$ and minimized the RMSE subject to f∈[0,1] and *S*.

#### 2.1.6. Membrane Perturbation

Lipid splays were defined as events when any of carbon atoms within lipid acyl chain was at least 2 Å further from membrane midplane than the phosphate atom of the same lipid. Lipids proximity to peptide was assessed based on the closest distance of their phosphate atoms to any heavy peptide atom. Lipids closer than 7 Å were considered as “close” and lipids further than 30 Å from peptides were considered for reference calculations. All results were block averaged, with block length of 50 ns, and the analysis was conducted for replicas simulated at 310 K.

Water membrane permeability, *P*, was estimated assuming inhomogeneous solubility-diffusion mechanism [46], based on water density profile across the membrane, ρ(z), and position dependent water diffusion coefficient in *z* direction, D(z):

(2) $$\frac{1}{P}=\int_{z_{1}}^{z_{2}}\frac{\rho_{0}}{\rho (z)D(z)}dz$$

with $\rho_{0}$ denoting bulk water density, and integral extending between ±40 Å from membrane center (we note that *P* was rather insensitive to integration interval as long as it encompassed membrane interior; hence, we resorted to such simple choice). Water density profiles were evaluated from tREMD runs for replica at T=310 K with gmx density tool. For the calculation of diffusion profiles tREMD trajectories were demuxed, and continuous trajectory fragments that remained at T=310 K for at least 10 ps were used for analysis. The *z* axis was discretized into bins, $z_{i}$ of 2 Å width and if a water molecule was found within a given bin at time *t*, i.e., $z\left(t\right)\in z_{i}$, its displacement within Δt=10 ps contributed to $D\left(z_{i}\right)=\langle {\left(z\left(t+\Delta t\right)-z\left(t\right)\right)}^{2}/2\Delta t\rangle$, where the averaging includes all such instances. To obtain final diffusion profiles for integration, $D(z_{i})$ were interpolated with a Gaussian kernel, with $\sigma_{D}=1$ Å dispersion. Water permeability obtained for pure POPC was $\left(9\pm 2\right)\times 10^{-3}$ cm/s, with error based on three simulation blocks (30 ns each), which is in fair agreement with experimental value of $\left(13.0\pm 0.4\right)\times 10^{-3}$ cm/s [47]. Water permeability in the presence of a peptide was calculated for the entire membrane patch and should be interpreted as corresponding to experimental conditions with 1:162 peptide to lipid ratio.

#### 2.1.7. Peptide Supervised Insertion

The procedure of peptide supervised insertion comprised 10 rounds of 50 simulations, 5 ns each. Starting structures included two random peptide placements and two selected from unconstrained simulations as already deeply inserted, all in hairpin conformation. After each round, the distance of W14 Cα atom from membrane surface (center of mass of phosphate atoms within given leaflet) was evaluated within the 4 last ns of each simulation, and a frame with maximum insertion depth was selected as a seed for subsequent round. The probability of reaching particular position within *i* rounds was evaluated as $p=\prod_{i}\frac{n_{i}}{50}$ with $n_{i}$ being the number of rounds in which the maximum insertion depth was within 0.7 Å from the global maximum.

### 2.2. Experimental Methods

#### 2.2.1. Materials

Materials were custom ordered with purity >95% (Lipopharm, Gdańsk, Poland). Sequences were as follows: wt GLFGAIAGFIEGGWQGMVDGWYGSGKKKKD and its E11A and W14A mutants. In all cases, the N-terminus was unmodified and the C-terminus was an amide. The -SGKKKD sequence was introduced to increase peptide solubility. Stocks were prepared from weighted amounts dissolved in water as 300–500 M solutions. Concentrations were checked by UV spectroscopy using the extinction coefficient at 280 nm of 12490 M${}^{-1}$cm${}^{-1}$ for wt and E11A peptides and 6990 M${}^{-1}$cm${}^{-1}$ for W14A. N-(7-Nitrobenz-2-Oxa-1,3-Diazol-4-yl)-1,2-Dihexadecanoyl-*sn*-Glycero-3-Phosphoethanolamine (NBD-PE) and Lissamine™ Rhodamine B 1,2-Dihexadecanoyl-*sn*-Glycero-3-Phosphoethanolamine (N-Rh-PE) used in fusion assays were from ThermoFisher Scientific. POPC (1-palmitoyl-2-oleoyl-*sn*-glycero-3-phosphocholine), Br${}_{4,5}$ (1-palmitoyl-2-stearoyl-(4,5)dibromo-*sn*-glycero-3-phosphocholine), Br${}_{6,7}$ (1-palmitoyl-2-(6,7-di-bromo)stearoyl-*sn*-glycero-3-phospho- choline), Br${}_{9,10}$ (1-palmitoyl-2-(9,10-dibro-mo)stearoyl-*sn*-glycero-3-phosphocholine), Br${}_{11,12}$ (1-palmitoyl-2-(11-,12-di-bromo)ste-aroyl-*sn*-glycero-3-phos-phocholine), and all other chemicals were from Sigma Aldrich (Merck, St. Louis, MO, USA). All experiments were performed in buffer pH 5.0 (10 mM citric acid/NaOH, 150 mM NaCl).

#### 2.2.2. Liposome Preparation

Desired amounts of lipids in chloroform were dried under a stream of nitrogen overnight under vacuum, followed by rehydration with appropriate buffer to 2–10 mg/mL concentration. For LUV preparation, the dispersion was frozen and thawed in liquid nitrogen and room temperature at least 5 times, followed by extrusion (15–21 times) through polycarbonate filters with 100 nm pores (Whatman) using Avanti Mini Extruder. For SUV preparation, the dispersion was sonicated with a tip sonicator (VibraCell VCX130) in 7–20 pulses lasting 10 s separated by 10 s breaks until the solution was clear.

#### 2.2.3. Lipid Mixing

Lipid mixing of membrane fusion was measured by FRET using a Cary Eclipse (Varian) spectrofluorometer. For each lipid composition, unlabeled and labeled LUVs were prepared. To prepare the labeled LUVs, we included 1 mol % NBD-PE and N-Rh-PE in the lipid mixture before drying the lipids in the liposome preparation procedure. Unlabeled and labeled LUVs were mixed at a 9:1 ratio in pH 5.0 buffer. The total lipids concentration was 0.2 mM. After the equilibration of the vesicles, an appropriate amount of peptides from a stock solution was added to give final concentrations of 4 M. Then, 10% Triton X-100 was added to achieve a final concentration of 1% after fusion had been completed. Fluorescence intensity of the acceptor (excitation with 463 nm and emission at 590 nm) before the addition of peptides and after the addition of Triton X-100 was defined as 0% and 100% fusion, respectively. Experiments were performed in triplicates and averaged signal is shown.

#### 2.2.4. Tryptophan Fluorescence

Fluorescence measurements were made with a Carry Eclipse (Varian) spectrofluorometer with an excitation wavelength of 280 nm, excitation/emission (2.4/4 nm), and photomultiplier voltage of 800 V. Spectra were measured using a 4 × 10-mm cuvettes (Hellma) in of 295–500 nm emission region with an increment of 1 nm. Peptide solutions were used in 2–10 M concentrations in 1000 L volume, titrated with increasing portions of SUV suspension up to 1 mM in 13–20 steps. Normally, for each lipid concentration, three spectra were averaged to achieve an adequate signal-to-noise ratio. The cuvette was in the contact with a thermostat, assuring constant temperature of 22.0 ± 0.5 °C. From each spectrum background was subtracted (by measuring blank titration). The titration curves were constructed as normalized intensity values for the wavelength for which the maximum spectral shift was observed between free and liposome-bound peptide (∼328 nm). Such procedure was shown to govern a linear response of the signal in respect to the titrated peptide [48]. The titration curves were further corrected for SUV scattering [48] by using the tryptophan (N-acetyl-L-tryptophanamide) fluorescence registered under the same conditions in the presence of SUV solution at concentration [L] according to the equation:

(3) $$F_{pept}^{corr}\left(\left[L\right]\right)=F_{pept}\left(\left[L\right]\right)\left(F_{Trp}^{buffer}\right)/\left(F_{Trp}\left(\left[L\right]\right)\right)$$

To corrected data point, non-linear hyberbolic curve was fitted according to the equation:

(4) $$F=1+\left(I-1\right)\left(K_{x}\left[L\right]\right)/\left(\left[W\right]+K_{x}\left[L\right]\right)$$

where I denotes asymptotic intensity value, [W] is the molar water concentration (55.55 M), and $K_{x}$ is the molar partition coefficient. Gibbs free energies were calculated as: $\Delta G_{x}^{\circ }=-RT\ln K_{x}$.

#### 2.2.5. Tryptophan Quenching

We measured the quenching of tryptophan residues inside lipid bilayers using lipids labeled with bromine at carbons 4–5, 6–7, 9–10 and 11–12 in the acyl chain (denoted as Br${}_{4,5}$, Br${}_{6,7}$, Br${}_{9,10}$, and Br${}_{11,12}$, respectively), with 20 mol% brominated PCs in place of POPC. The same spectral conditions and spectrofluorometer settings were used as in binding experiments. Aliquots of peptide solution were added to vesicle suspensions to achieve a final peptide/lipid ratio of 1:500 and incubated for 2 min before recording tryptophan fluorescence intensity. The signal from an identical sample without peptide was used to determine the quenching ($F_{m}/F_{0}$).

### 2.3. Leakage Assay

Leakage assay was performed by monitoring fluorescence increase of calceinrealeased from LUV interior. Calcein was encapsulated in LUVs by hydrating lipid film by dye solution (112 mM of calcein in 0.27 M NaOH). Free dye and liposomes were separated to fraction by size exclusion chromatography (PD-10 columns, GE Healthcare). The most concentrated fractions with liposomes were pooled. Final concentrations of lipids were 0.625 mg/mL. Fluorescence was recorded with Carry Eclips (Varian) using single excitation/emission wavelengths (495/518 nm). Fluorescence baseline of 3.65 M liposomes was observed for 2 min (subsequently rescaled to 0% leakage). Then, the peptides were added to the solution at 1:50 peptide/lipid ratio and the leakage was measured over 4000 s. The remaining intact liposomes were disrupted by adding 10 L of 10% Triton X-100 to the solution (100% leakage).

## 3. Results

### 3.1. Peptide Configurations

To investigate HAfp configuration space and its alteration by two known function-affecting mutations, we conducted a set of tREMD simulations for surface and deeply inserted peptides. Taking into account structures based on experimental data, the simulations of surface bound peptides started from all hairpin or all boomerang structures. In turn, all deeply inserted peptides were initially modeled in hairpin conformation, based on our previous results obtained using the self assembly technique [20].

#### 3.1.1. Surface Placement

**Figure 1:**
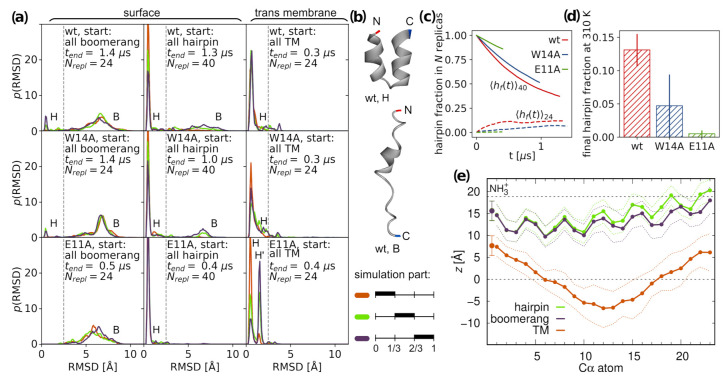
(**a**) Time evolution for distributions of backbone atoms RMSD with respect to tight helical hairpin (NMR structure, PDB id 2kxa, model 1) at 310 K from tREMD simulations. (**b**) Representative conformations for two most populated states. (**c**) Time evolution of hairpin fraction in tREMD simulations based on fitted kinetic equations. (**d**) Equilibrium hairpin fractions at 310 K. (**e**) Insertion depths of N-terminal amino group nitrogen and Cα atoms of wt HAfp ± one standard deviation (dotted lines). Dashed lines are the membrane surface (the maximum of phosphate atoms density).

Regardless of initial configurations, surface bound peptides started to interconvert between tight helical hairpin and open boomerang forms, as is apparent based on the gradual formation of bimodal RMSD distributions (Figure 1a). Representative structures, obtained in each case as the centroids of the most populated conformation clusters, correspond well to experimentally determined boomerang and hairpin geometries, with minor differences between the considered peptide versions (Figure 1b and Figure S1). The insertion depth of surface configurations turns out to be the same for each variant (Figure 1e and Figure S1) and displays only slight conformation-dependent alterations. In general, the N-helices do not change their position relative to membrane surface, irrespective of hairpin opening or closing, while the C-helices of boomerang configurations are slightly more deeply inserted.

Given the adopted tREMD protocol and relatively small size of the peptides under study, we aimed to determine equilibrium populations of surface conformations in each case. Unfortunately, despite the long accumulated sampling time (85, 73, and 28 s for wt, W14A, and E11A, respectively), the RMSD distributions were still far from equilibria, indicating rather slow conformational dynamics on membrane surface. To estimate the final proportion of closed and open configurations, we assumed that the transition between them is a two-state process and considered a set of temperature-dependent kinetic equations operating on stochastic tREMD trajectories (Figure 1c) [41]. The extrapolation of fitted solutions to infinite time for tREMD simulations starting from all hairpin, and, likewise, all boomerang structures should, in principle, lead to the same equilibrium hairpin population at T=310 K. Accordingly, we estimated this population as an average from both kinds of tREMD runs and obtained 0.13±0.02, 0.05±0.05, and 0.005±0.005 hairpin fractions for wt HAfp, W14A, and E11A mutants, respectively, at T=310 K (Figure 1d). In qualitative agreement with experimental data, this result indicates greater preference towards hairpin structure for wt HAfp compared to W14A mutant [11,14]. We did not find direct experimental evidence for the nature of the dominant E11A conformation, but, based on its reported thermodynamic binding signature with moderately unfavorable entropy component similar to W14A and rather different from highly entropy penalized wt HAfp [14], the dominance of less constrained boomerang structure is likely also in its case.

Our predicted population of 0.13 closed structures for wt HAfp23 is considerably lower compared to estimates based on NMR in DPC micelles that suggest exclusively hairpin conformation at neutral pH, exemplified by 2kxa PDB structure [11]. In the same system, but at pH = 4, to which our simulation conditions are adjusted (protonated E11), closed conformations were found to constitute 0.8 of the entire population and were estimated to interchange with the open ones on ∼25 s time scale [49]. Furthermore, as compared to detergent micelles, the closed form is apparently less stable in membranes, where its population of 0.7 was reported already at neutral pH [50]. Taken this into account, as well as the fact that sole W14A substitution is enough to permanently open up the hairpin, it is plausible that the stabilization of the closed structure is not very strong, and that its interconversion with non-negligible boomerang fraction indeed occurs in POPC membranes for wt HAfp.

#### 3.1.2. Intramembrane Placement

**Figure 2:**
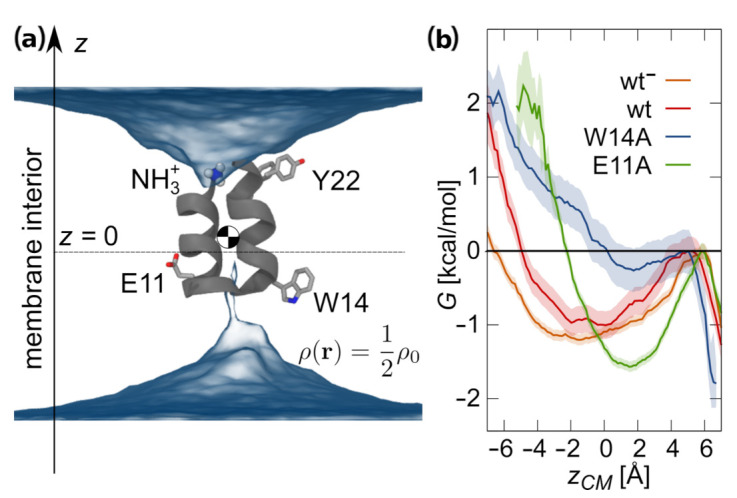
(**a**) Representative membrane-spanning configuration of wt HAfp with average water density isosurface at half of bulk density. (**b**) Free energy profiles for unrestrained peptides center of mass in membrane-spanning configurations.

Although shorter than typical transmembrane helical fragments, HAfp in hairpin conformation may be capable of adopting intramembrane position [20]. It can do so by inducing local membrane thinning that provides hydration of both hydrophilic hairpin poles necessary for the stabilization of the membrane-spanning orientation (Figure 2a). In the case of wt HAfp, the deeply inserted hairpin is located centrally within the membrane, with residues 6 and 18 positioned at the bilayer midplane (Figure 1e). Notably, the nuclear Overhauser effect between peptide amide groups and terminal methyl groups of lipid acyl chains was observed exactly and exclusively for these two residues in the study of wt HAfp23 in DPC micelles [11]. Given relatively shallow insertion depths observed in simulations of open structures (Figure 1e), this would be not possible at all without assuming intramembrane configurations.

Hairpin conformation appears to be well preserved for deeply inserted peptides (Figure 1a), although we observed solitary unfolding events that lead to surfacing of the C-terminal arm at the opposite membrane side to the N-terminus—we note, however, that this would be impossible, if the peptide was attached via a linker to the remaining part of the HA2 subunit. Our simulations indicate that the intramembrane configuration is generally metastable, and can be abandoned by peptide escaping to the surface in the direction of hairpin opening. We observed no successful, complete egress in the opposite direction (i.e., towards the hairpin apex) in any of our simulations, likely due to firm anchoring of the positively charged N-terminal amino group within the aqueous compartment.

The stability of intramembrane localization appears to be affected by amino acid composition of the peptide. Wt HAfp remains most deeply inserted among the considered variants, and is separated from surface configuration by ∼1 kcal/mol free energy barrier (Figure 2b). Intriguingly, the deprotonation of E11, which may be plausible owing to the exposure of hairpin apex to the neutral pH of cellular environment on the outer side of the endosomal membrane, further deepens and stabilizes peptide insertion. The W14A mutant is least stable, with practically no free energy barrier for surfacing, and roughly 3 Å shallower insertion depth compared to wt HAfp. We attribute this behavior to the decrease of hairpin apex hydrophilicity upon the removal of W14 side chain. In turn, E11A substitution leads to intermediate insertion depth, albeit relatively well stabilized within the membrane core. In this case, in contrast to other variants, a partial remodeling of the C-terminal hairpin arm is also observed (Figure S1).

To check whether the stability of intramembrane configurations is not merely a spurious effect of the selected Amber force field family, we carried out an independent set of tREMD simulations for wt using unrelated Charmm force field (peptide and lipids). We observed a stable, membrane-spanning hairpin, with even greater free energy barrier for surfacing of ∼2.5 kcal/mol than in the Amber force field (Figure S5).

### 3.2. Potentials of Mean Force

**Figure 3:**
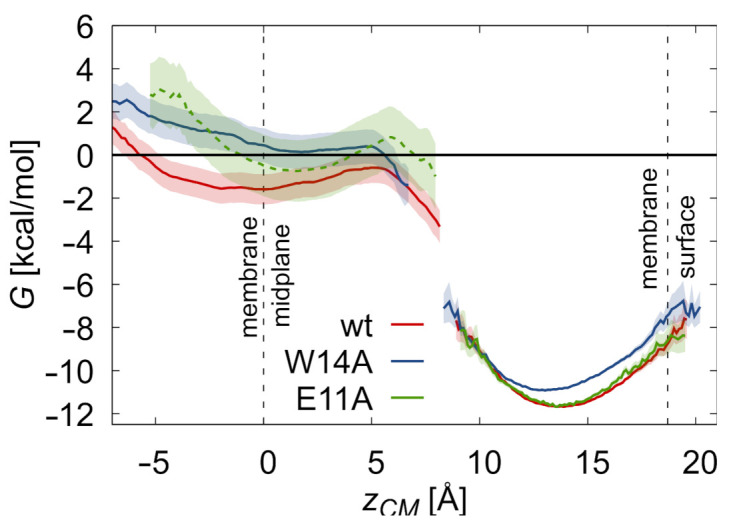
PMF for unrestrained peptides in deep and surface free energy minima. G=0 corresponds to free peptides in the aqueous phase. Relative depths of free energy wells for surface and deep configurations were obtained based on: continuous PMF for restrained peptides, (un)restraining free energy calculations, and free peptides simulations (Table 1 and Figure S4). Dashed line for E11A reflects the fact that its surface minimum was estimated based on binding free energy for wt.

To investigate peptides partitioning between membrane surface and interior, we evaluated free energy differences between the respective configurations (Figure 3). To this end, we took into account the free energy cost of peptide restraining to hairpin conformation at membrane surface assuming hairpin to boomerang ratios summarized in Figure 1d, the potential of mean force (PMF) for restrained hairpin translocation into the membrane core, and free energy gain upon the release of the restraining potential thereof. To further complement the calculations, we assessed the cost of hairpin unbinding from surface and unfolding in bulk solvent (for details, see the Supplementary Materials).

The calculated free energies of peptides binding to membrane surface (Table 1, $\Delta G^{B\to S}$) are in the order of 11 kcal/mol. They do not include, however, the effect of peptide liberation from a fixed position in the bulk, which should generally decrease the calculated affinities by 1–2 kcal/mol, depending on the assumed free peptide concentration. Our estimate of the wt HAfp binding strength is ∼1 kcal/mol above that of the W14A mutant, which is in fair agreement with our own measurements (Table 1, $\Delta G_{exp}$, Figure S9) and other published data [14]. Free energy profiles for further peptide transition from membrane surface towards the core rise steeply in all three considered cases, and barriers leading to relatively shallow intramembrane minima are achieved when peptides centers of mass are around 6 Å from bilayer midplane (Figure 3). The estimated free energy cost of peptide transitioning from membrane surface to the core, $\Delta G^{S\to D}$, is 10 kcal/mol for the wt HAfp and 11 kcal/mol for both mutants. The overall transition cost from surface to deep minimum is a combination of three effects. The first is the cost of hairpin restraining on membrane surface, $-\Delta G_{h\to 0}^{S}$. Here, naturally closed peptides have an advantage over those assuming predominantly open conformation, as is reflected by almost 3 kcal/mol difference between wt HAfp and E11A.

The second, is the cost of restrained hairpin translocation to intramembrane position. Somewhat surprisingly, the results for wt HAfp and W14A mutant are quite similar in this respect (see Figure S3 for full, detailed PMF). It indicates that, instead of changing intrinsic peptide propensity to move from membrane surface to the core, W14A mutation rather affects peptide behavior within both free energy wells by promoting the open surface form and weakening the stability of the membrane-spanning configuration. In turn, membrane penetration of the restrained E11A mutant is least energetically costly, most likely owing to increased hydrophobicity of the hairpin apex, that is the peptide part that actually penetrates through the core. The third free energy contribution, $\Delta G_{h\to 0}^{D}$, comes from the removal of the restraining potential for membrane-spanning configurations. We do not see large differences here between the considered HAfp versions. In all cases, free energy gain is smaller than the corresponding effect on the surface, indicating greater hairpin rigidity within the membrane core.

Taken together, the overall cost of peptide translocation into the membrane core appears to be rather high and, based on the considered simulation setup, indicates that the population of deeply bound peptides is very small. Nevertheless, the order wt > E11A > W14A observed both for the availability and stability of membrane-spanning configurations correlates well with peptides fusogenic activities (Figure S8), suggesting a hypothesis that deep insertion may be relevant to their mechanism of action.

### 3.3. N-Helix Orientation

**Figure 4:**
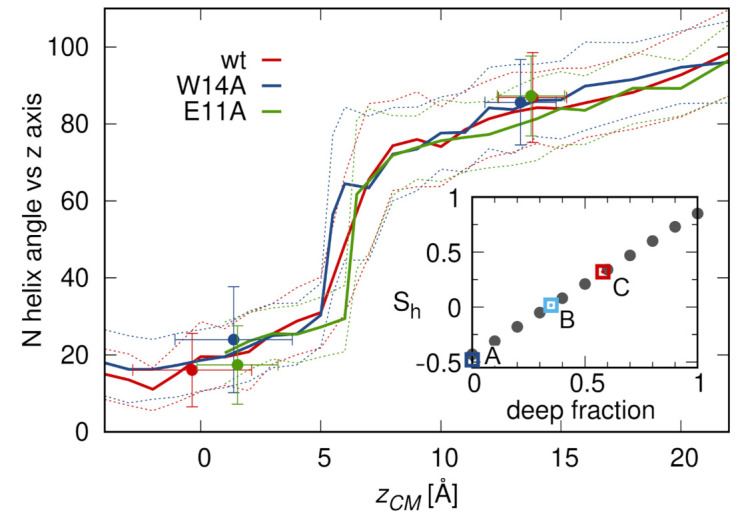
N-helix angle with respect to membrane normal in degrees as a function of insertion depth. Lines represent data from umbrella sampling simulations for restrained hairpin conformations, while points are data from unrestrained simulations. Error bars correspond to one standard deviation. Inset: The estimated N-helix order parameter as a function of deep configurations fraction. Squares are the experimental data for $S_{h}$: (A) W14A [14]; (B) wt HAfp20 [53]; and (C) wt HAfp23 [17,18,52].

Experimental evidence concerning peptide orientation within the membrane often comes from ATR-FTIR spectroscopy [51]. Assuming the dominance of helical structure, at least within the N-helix, the obtained order parameter, $S_{h}$, allows determination of the average angle between helix axis and membrane normal, *z*. In this respect, $S_{h}$ for an at least 23 residue wt HAfp is reported to be in the range from 0.32 to 0.59, depending on pH and membrane composition [17,18,52], which is interpreted as an oblique helix insertion, with 30–50 degrees average tilt with respect to the *z* axis. A similar oblique insertion angle of 50 degrees was suggested based on EPR measurements [10]. Our simulations do not support this view. In all surface configurations, the N-helix remains parallel to membrane plane (Figure 1e and Figure 4), and its capping NH${}_{3}^{+}$ group does not reveal any tendency for deep membrane penetration. On the contrary, if the peptides are forced to move into the membrane during umbrella sampling runs or supervised insertions (see below), they penetrate the hydrophobic core exclusively with the hairpin apex. In doing so, however, they do not assume any intermediate, oblique orientations, but rather shift sharply from parallel to almost perpendicular orientation upon passing the free energy barrier between surface and deep configurations (Figure 4).

This simulation based picture suggests a two states contribution to the order parameter. If we calculate $S_{h}$ with the assumption of bimodal tilt angle distribution taken from simulation data, the experimental values for wt HAfp23 are recovered for 0.5–0.6 fraction of deep configurations (Figure 4, inset). This fraction would be lower for HAfp20 (reported $S_{h}=0.18$ [53], deep fraction 0.4) and nonexistent for the W14A mutant (reported $S_{h}=-0.48$ [14]), in agreement with surface only configurations in this case.

### 3.4. TRP Fluorescence

To further confront our simulations with experimental findings, we analyzed depth-dependent tryptophan fluorescence quenching by brominated lipids [54]. Here, the decrease in TRP fluorescence intensity with respect to a reference value, $F/F_{0}$, caused by Br atoms located at known positions along lipid acyl chains reveals an approximate insertion depth of TRP side chains. Assuming distance-dependent model of fluorescence quenching (see Section 2.1.5 for details) [54] and applying it to peptide-lipids configurations from our tREMD simulations, we assessed what combination of major macrostate populations: surface hairpin, surface boomerang, and deeply inserted hairpin, best reproduces experimental data.

**Figure 5:**
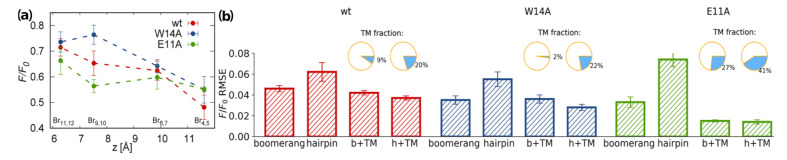
(**a**) Experimentally determined fluorescence quenching ratio, $F/F_{0}$, for peptides in brominated lipids; and (**b**) RMSE values for best fit between experimental and calculated $F/F_{0}$. Circle plots indicate the fraction of transmembrane configuration providing the lowest RMSE.

For all three considered peptides, the measured quenching effect is strongest for Br probes that are closest to bilayer surface (Br${}_{4,5}$) and generally decreases towards membrane interior (Figure 5a). Importantly, however, the readouts differ between peptide mutants, indicating higher fluorescence quenching by deeply buried probes in the case of the E11A mutant and wt HAfp, compared to the W14A mutant. In all cases root mean square errors (RMSE) between experimental $F/F_{0}$ ratios and predictions based on MD simulations clearly disfavor standalone surface hairpin configurations (Figure 5b). Since for the wt HAfp the hairpin is indicated as a dominant structure by NMR [11], it suggests the need to include deep configurations in order to explain fluorescence data. Indeed, 20% of deep configurations in addition to surface hairpin leads to the overall lowest RMSE in this case. For the W14A mutant, the hairpin structure is unlikely [14], which leaves surface boomerang in best agreement with fluorescence data. Notably, in this case, the inclusion of deep configurations does not improve RMSE. On the contrary, favorable agreement for the E11A mutant clearly requires the assumption of ≳30% of intramembrane locations, but the results do not discriminate between boomerang and hairpin configurations.

The results unanimously suggest a greater tendency to penetrate membrane interior for the wt HAfp and E11A mutant compared to the W14A mutant. While there is certainly no quantitive match between population fractions assessed from the PMF calculations and the estimates based on TRP fluorescence quenching or ATR-FTIR spectroscopy discussed above, we note that the simulation model most likely overestimates the free energy difference between surface and deep minima due to limited system size that enhances the compactness of the considered membrane slab, but it still captures qualitative differences between the considered peptide variants.

### 3.5. Membrane Perturbation

#### 3.5.1. Lipid Tail Protrusions

**Figure 6:**
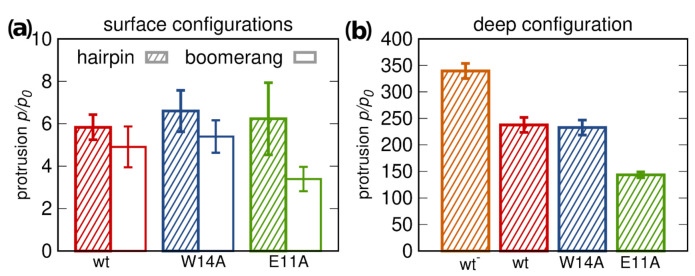
Ratios of lipid tail protrusions within 7 Å distance from peptides to those in pure POPC membrane for: (**a**) surface; and (**b**) deep configurations.

The extent of peptide-promoted lipid tail protrusions [30] has often been invoked as a predictor of their ability to induce lipid mixing between membranes, as a proxy for fusogenic activity [23]. According to our simulations, the presence of surface bound peptides indeed increases the likelihood of lipid tail protrusions, but only roughly sixfold compared to pure POPC membrane (Figure 6a). Aside from being relatively weak and showing little dependence on peptide conformations, the effect observed for surface configurations is equally strong for fusion active wt HAfp and inactive W14A mutant, and, hence, does not explain the loss of function in this latter case.

The situation changes, if membrane-spanning configurations are considered. Firstly, they appear to be capable of increasing the intensity of lipid tail protrusions roughly 250 fold compared to pure membrane (Figure 6b). It is most likely a consequence of deeper insertion of the positively charged N-terminal amino group (Figure 1e and Figure S2), which allows more effective dragging of phosphate groups below membrane surface, thus promoting lipid tilting and splaying. Secondly, the assumption of only surface bound configurations for the W14A mutant, inferred from previous results, would explain its inactivity. Thirdly, comparably lower protrusion promoting capability of the E11A mutant compared to the wt HAfp corresponds well to their relative fusogenic activity. The difference is possibly related to shallower insertion depth the E11A mutant, and consequently lower burial of the N-terminal peptide charge (Figure S2). Finally, of note is the highest rate of lipid protrusions generated by the wt${}^{-}$ HAfb with E11 deprotonated, whose occurrence in real life scenarios cannot be excluded given the exposure of the hairpin apex to cellular environment.

#### 3.5.2. Membrane Water Permeability

To further assess membrane perturbation induced by the peptides, we calculated relative membrane water permeability and compared it with experimental leakage assays of LUV encapsulated calcein.

**Figure 7:**
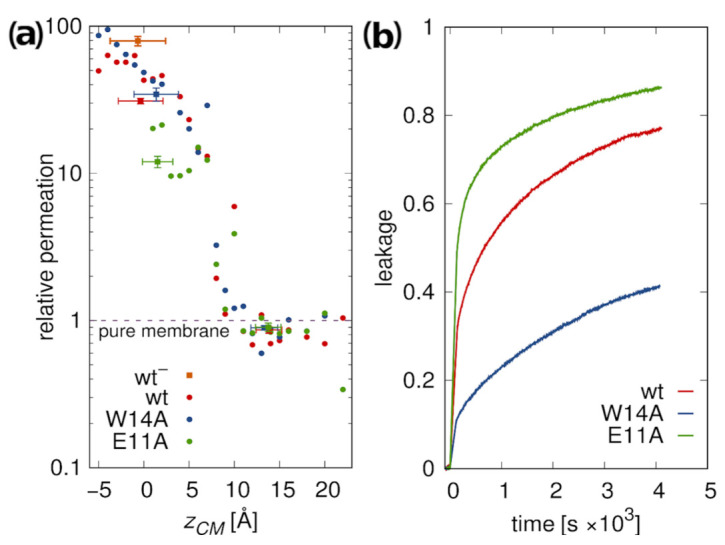
(**a**) POPC membrane water permeation for 1:162 peptide to lipid ratio, relative to pure membrane slab composed of 162 lipids. Dots correspond to values obtained from umbrella sampling simulations for restrained hairpin conformations, squares to values obtained from unrestrained simulations. (**b**) Peptide-induced calcein leakage from POPC LUVs.

According to simulations, peptides located at the membrane surface do not lead to any increase in its water permeability compared to pure POPC membrane (Figure 7a). However, a sharp onset of permeability is observed upon transition to deep minimum. This effect is somewhat less pronounced for the E11A mutant, possibly due to its greater hydrophobicity and smaller insertion depth. The highest permeation was observed for the most deeply inserted wt${}^{-}$ variant. The simulated results are in fair agreement with the experimental leakage assays (Figure 7b). Apparently lower leakage caused by W14A mutant as compared to other peptides may be explained by its less stable deep configuration (Figure 3). Notably, however, relative magnitudes of leakage for the wt HAfp and E11A mutant observed in experiments are opposite to those suggested by simulations. In agreement with the interpretation of fluorescence quenching experiment (Figure 5b), this may indicate that the fraction of deeply inserted E11A configurations may be actually somewhat greater than in the case of wt. Taken together with greater stability but shallower depth of E11A transmembrane free energy minimum (Figure 2b), this would imply that comparably greater fusogenic activity of the wt is predominantly the effect of its ability to localize deeper within the membrane core. To check whether the difference between W14A and wt/E11A peptides was not driven by a diminished surface concentration resulting from an order of magnitude lower partition coefficient for W14A (∼1.6 kcal/mol difference in Gibbs binding free energy, Table 1), we also performed the experiment at lower (1:500) peptide/lipid ratio. All three leakage curves looked more similar to each other; however, wt and E11A peptide still led to more efficient permeation (Figure S10).

### 3.6. Supervised Insertion

As indicated by our PMF calculations, the peptides need to traverse a substantial free energy barrier of roughly 10 kcal/mol in order to reach membrane interior from surface configurations. To check whether such putative transitions are feasible for unconstrained peptides and to assess their time scale, we devised a supervised insertion scheme. It is based on consecutive simulation rounds, each including relatively short (5 ns) multiple (50) unconstrained MD runs. After each round, a structure that is most advanced along the assumed pathway is selected among all runs and is used as a seed for a subsequent round of simulations. We focussed on wt HAfp and considered the position of W14 Cα atom along the *z* axis as a measure of insertion progression.

**Figure 8:**
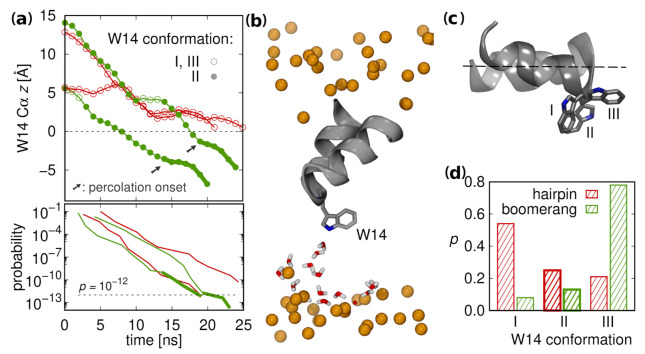
(**a**) (Top) Supervised insertion trajectories for two successful (green) and two unsuccessful trials (red); and (Bottom) the probability of insertion advancing along *z* axis. (**b**) Water wire established by interaction with W14 at percolation onset (trajectory snapshot). The water molecules within 10 Å of protein atoms and *z* coordinates restricted to protein-distal membrane leaflet are shown. The orange spheres represent membrane phosphate atoms. (**c**) Three main W14 conformations. (**d**) Probability distribution for finding W14A conformers in surface configurations.

Of four independent supervised insertion procedures initiated from diverse starting configurations two turned out to be successful in peptide reaching the deep free energy well within 10 simulation rounds (Figure 8a, Top). In each such case, prior to achieving membrane-spanning configuration, the peptide induced the formation of a thin water wire between W14 side chain and aqueous compartment on the opposite membrane side (Figure 8b). The apparent role in this process was played by W14 indole nitrogen atom. It was effective, however, only in one of three possible side chain conformers that provided cross-membrane facing nitrogen orientation (Figure 8c), and without assuming this conformer peptide progression was stalled. Intriguingly, this conformer appears more frequently among closed rather than open surface configurations (Figure 8d), thus contributing further to hairpin insertion readiness, in addition to its already more favorable shape. To test an alternative hypothesis of N-terminus driven insertion, we also carried out simulations based on supervised terminal amino group nitrogen atom position, but none of the four trials for hairpin structure was successful (Figure S6).

The time scale of complete peptide insertion was roughly 20 ns. By simply evaluating the probability of completing the entire route as a product of ratios of productive to unproductive runs in each of 10 rounds (see methods for details), we obtain the probability of success in a single attempt, $p∼10^{-12}$ (Figure 8a, bottom). Following a coarse reasoning, for a GUV with 10 μm diameter and 160 lipids to peptide ratio, this gives an estimate of >150 insertions per second, per vesicle, thus approaching the time scales of experimental fusion observation.

### 3.7. Possible Insertion Modes

**Figure 9:**
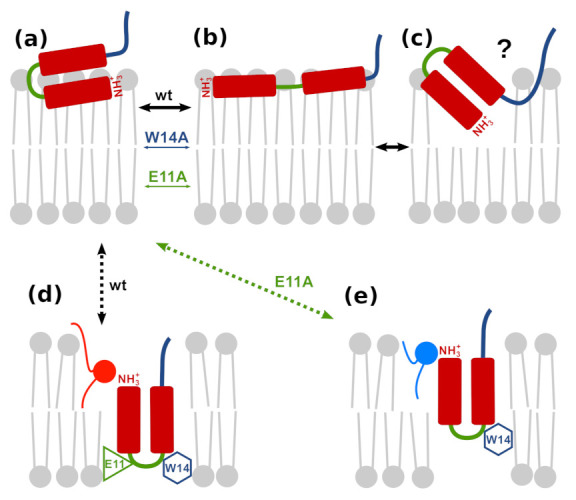
Possible insertion modes of HAfp into lipid membrane: (**a**,**b**) surface hairpin and boomerang, respectively; (**c**) obliquely inserted hairpin; (**d**) deeply inserted transmembrane hairpin (wt); and (**e**) shallowly inserted transmembrane hairpin (E11A).

The dominant view of wt HAfp23 configurations based on experimental insights to date corresponds to a tightly closed hairpin [11] which remains partially inserted into membrane, with buried N-terminus and solvent-exposed kink, such that the overall tilt angle of the N-helix with respect to membrane normal is ∼40 degrees (Figure 9c) [10,14]. We do not find support for this view in our simulations. We also note that such configuration would entail at least partial burial of the C-terminal hairpin fragment (together with a few residues from subsequent linker region, if complete HA structure was considered) whereas its strictly conserved sequence, ${}^{21}$WYG${}^{23}$, suggests rather a preference to the membrane–water interface region. Instead, we propose a more complex model, in which the peptides fluctuate between closed and open surface conformations (Figure 9a,b) and occasionally dive into the membrane core, adopting more or less stable transmembrane configuration (Figure 9d,e). In this view, the fusogenic HAfp activity would be related either to this latter configuration alone or to membrane-perturbing transitions of buried hairpins back to membrane surface.

## 4. Discussion

Our simulations indicate the highly dynamic nature of HAfp, which includes interconversions between open and closed surface conformations, as well as transient excursions into the membrane core. The lack of notable differences in the level of membrane perturbation by surface configurations of active and inactive mutants combined with apparently greater ability of the active ones to adopt transmembrane placement with highly membrane disruptive potential indicate that peptides’ ability to dive into the membrane core may be the key element of their fusogenic function.

Indeed, experimentally observed activity of the three considered HAfp versions appears to correlate with the stability and insertion depth of their membrane-spanning configurations. According to our results, the actual HAfp fusogenic mechanism is related to partial burial of positively charged N-terminal amino group whose interaction with membrane phosphates promotes lipids tilting and, eventually, acyl tails protrusions. While such role of the N-terminal charge has already been postulated, its burial was ascribed to oblique peptide insertion whose existence is not supported by our study. Instead, we advocate that the experimental measurements consistent with such oblique insertion reflected an average over two, distinct states corresponding to in-plane and transmembrane orientations. While at first sight the relatively short length of HAfp helical hairpin arms appears incompatible with transmembrane configurations, in fact, it is well suited to provide for efficient burial of the N-terminal amino group within bilayer core. An extension of the N-helix would lead to surfacing of the N-terminus and a stable, yet benign membrane-spanning structure, typical for integral membrane proteins. Instead, the necessity to maintain a metastable deep configuration by keeping balanced interaction with aqueous compartment on both membrane sides imposes strict requirements for the kink region that likely explain the conservation of residues that support tight packing of both helices, such as the glycine ridge, as well as the existence of the essential W14, whose role in membrane penetration and water attraction was highlighted by our simulations.

The free energy difference between surface and deep configurations resulting from our simulations is large and suggests that the fraction of deeply inserted peptides may be rather small. We note, however, that our free energy calculations likely provide an upper limit to the actual value due to relatively small size of the membrane patch considered in our model. In reality, deep peptide insertions may be happening opportunistically supported by local membrane fluctuations. However, even rare such events yet of relatively high fusogenic potential are enough to explain the course of experimentally observed vesicle fusion, whose time scale extends to minutes.

## 5. Conclusions

Our study leads to the following conclusions:

- A model assuming the presence of HAfp exclusively at lipid–water interface is not sufficient to explain mutation-related differences in peptides activity, experimentally estimated helix tilting angle, or peptide-induced membrane water permeability.
- Simulations together with tryptophan fluorescence quenching experiments and the observation of peptide-induced membrane water permeability suggest the existence of membrane-spanning configurations with high membrane-perturbing potential.
- The effect of amino acids mutations on fusogenic activity correlates with peptides ability to achieve and maintain deeply inserted configurations and with the insertion depth of the N-terminal amino group.
- Although surface HAfp binding seems to be the dominant mode, simulations demonstrate the feasibility of spontaneous deep peptide insertions at sufficient rate to promote fusion in experimentally observed time scale.

## Figures and Tables

**Table 1:** Experimental, $\Delta G_{exp}$, and calculated free energy differences in kcal/mol (error estimates in subscript): $\Delta G^{B\to S}$ binding from bulk solvent to membrane surface, $\Delta G^{S\to D}$ transition from surface to deep configuration, $\Delta G_{0\to h}^{B}$ the cost of peptide restraining to hairpin in bulk solvent, $\Delta G_{h}^{B\to S}$ transition of restrained peptide from bulk solvent to membrane surface, $\Delta G_{h\to 0}^{S}$ restraints removal for surface configuration, $\Delta G_{h}^{S\to D}$ transition of restrained peptide from surface to deep configuration, $\Delta G_{h\to 0}^{D}$ restraints removal for deep configuragion. ${}^{*}$ experimental energies are for peptides with solubility tags, and † value for E11A based on experimental difference wrt. wt.

Peptide	$\Delta G_{\exp}^{*}$	$\Delta G^{B\to S}$	$\Delta G^{S\to D}$	$\Delta G_{0\to h}^{B}$	$\Delta G_{h}^{B\to S}$	$\Delta G_{h\to 0}^{S}$	$\Delta G_{h}^{S\to D}$	$\Delta G_{h\to 0}^{D}$
wt	$-10.2_{0.1}$	$-11.7_{0.9}$	$10.1_{0.7}$	$18.3_{0.3}$	$-20.0_{0.7}$	$-9.9_{0.2}$	$8.6_{0.3}$	$-8.5_{0.2}$
W14A	$-8.6_{0.1}$	$-10.9_{1.1}$	$11.3_{0.8}$	$17.9_{0.6}$	$-18.6_{0.6}$	$-10.3_{0.2}$	$8.5_{0.3}$	$-7.7_{0.1}$
E11A	$-10.3_{0.1}$	$-11.8_{0.9}^{†}$	$11.0_{1.4}$	−	−	$-12.6_{1.2}$	$6.5_{0.2}$	$-8.5_{0.2}$

## Data Availability

MD configuration files, system topologies, system structures, trajectories, and source experimental data are available on figshare (DOI 10.6084/m9.figshare.14365508).

## Supplementary Materials

The following are available online at https://www.mdpi.com/article/10.3390/ijms22105301/s1, Figure S1: Representative conformations from unrestrained simulations for three peptide variants and the distribution of root mean square deviation from tight hairpin structure (PDB. 2kxa, model 1) for wild type, E11 deprotonated hemagglutin fusion peptide (wt${}^{-}$ HAfp) simulation in trans-membrane orientation. Figure S2: Insertion depths of N-terminal amino group nitrogen atom and Cα atoms of wt HAfp in considered configurations. Figure S3: Potentials of mean force for translocation between bulk water and membrane center for restrained peptides. Figure S4: Relations between contributions to free energy considered for obtaining binding free energies of unrestrained peptides at surface and deep minima, relative to unrestrained peptides in water. Figure S5: Results for Charmm36 force field. Figure S6: Supervised insertion attempts based on N-terminus progression. Figure S7: Scheme for Trp quenching calculations. Figure S8: Experimental results for lipid mixing. Figure S9: Experimental results for peptide binding to POPC SUVs. Figure S10: Experimental results for calcein leakage. Table S1: Insertion depths of Br atoms. Table S2: List of tREMD temperatures and list of conducted simulations. Table S3: List of umbrella sampling runs.
