# Supplementary material for: Transient Excursions to Membrane Core as Determinants of Influenza Virus Fusion Peptide Activity
Source: Int J Mol Sci. 2021 May 18;22(10):5301. doi: 10.3390/ijms22105301 (PMC8157580; doi:10.3390/ijms22105301)
Supplement: Supplementary file 1 [file ijms-22-05301-s001.zip › ijms-1205057-supplementary.pdf]

Transient excursions to membrane core as determinants of influenza virus fusion peptide activity

Supporting Information

Remigiusz Worch[1], Anita Dudek[2], Paulina Borkowska[1], and Piotr Setny[2,*]
[1]Institute of Physics, Polish Academy of Sciences, Aleja Lotnikow 32/46, PL-02668 Warsaw, Poland
[2]Centre of New Technologies, University of Warsaw, Banacha 2c, 02-097, Warsaw, Poland
*p.setny@cent.uw.edu.pl

Representative structures from tREMD simulations and results for E11 deprotonated peptide (wt$^-$)

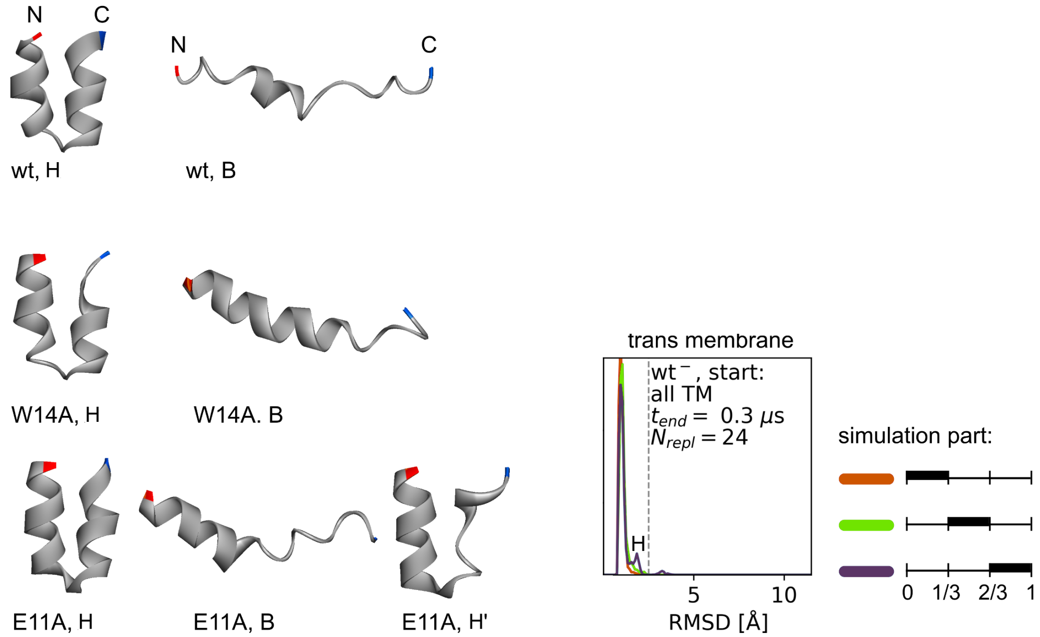

Figure 1: Left: representative conformations from unrestrained simulations for 3 peptide variants. Right: the distribution of root mean square deviation from tight hairpin structure (PDB. 2kxa, model 1) for wild type, E11 deprotonated hemagglutin fusion peptide (wt$^-$ HAfp) simulation in transmembrane orientation.

Insertion depths of peptide atoms

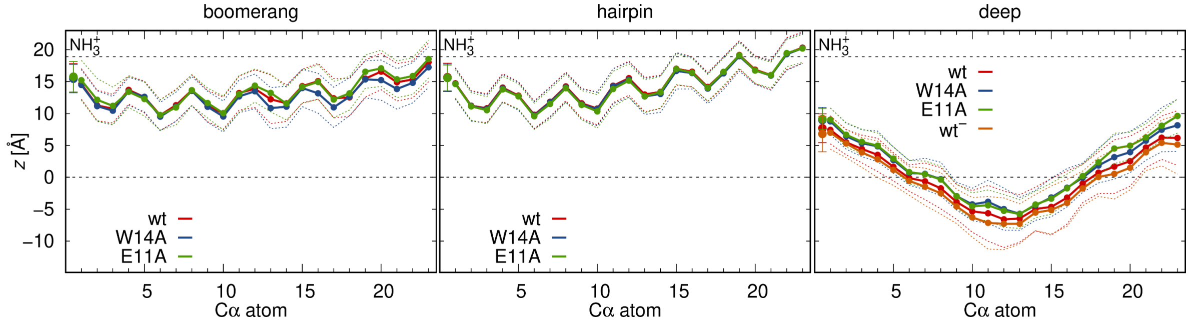

Figure 2: Insertion depths of N-terminal amino group nitrogen atom and Cα atoms of wt HAfp in considered configurations. Dotted lines correspond to $\pm$ one standard deviation. Dashed lines denote membrane surface (the maximum of phosphate atoms density) at $z = 18.9$ Å, and mid-plane at $z = 0$.

Free energy profiles and free energy calculations.

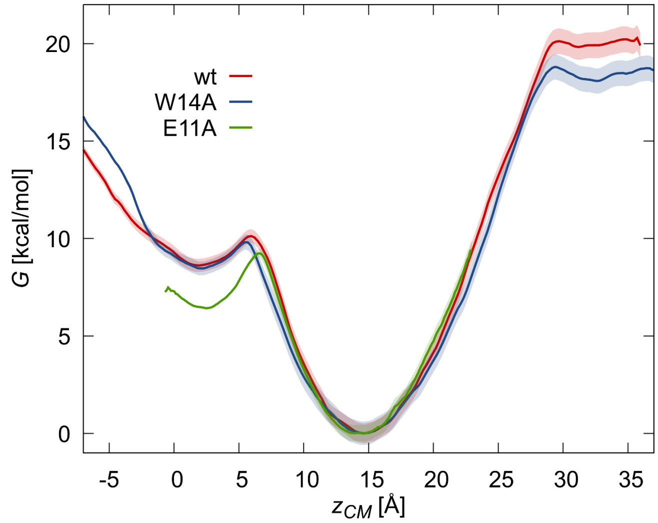

Figure 3: Potentials of mean force for translocation between bulk water and membrane center ($z = 0$) for three peptide variants in helical hairpin configurations enforced by restraining the distances between Cα atoms to those observed in HAfp$_{1-23}$ NMR structure (pdb 2kxa, model 1). All curves were shifted so that $G = 0$ corresponds to the minimum of surface free energy basin.

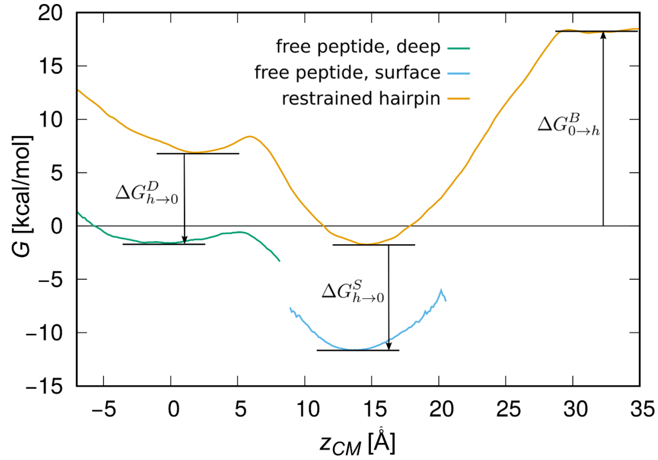

Figure 4: Relations between contributions to free energy considered for obtaining binding free energies of unrestrained peptides at surface and deep minima, relative to undestrained peptides in water. The curves represent data for wt. Free energy level for restrained hairpin in bulk water was assessed based on averaging of PMF from 31 to 35 Å. Free energy levels for free energy basins were taken as local PMF minima. Peptide binding free energies to respective basins were calculated as follows: a) binding from bulk solvent to membrane surface $\Delta G^{B\to S} = \Delta G^{B}_{0\to h} + \Delta G^{B\to S}_{h} + \Delta G^{S}_{h\to 0}$, b) transition from bulk surface to deep minimum: $\Delta G^{S\to D} = -\Delta G^{S}_{h\to 0} + \Delta G^{S\to D}_{h} + \Delta G^{D}_{h\to 0}$, with the following meaning of free energy contributions: $\Delta G^{B\to S}$ binding from bulk solvent to membrane surface, $\Delta G^{S\to D}$ transition from surface to deep configuration, $\Delta G^{B}_{0\to h}$ the cost of peptide restraining to hairpin in bulk solvent, $\Delta G^{B\to S}_{h}$ transition of restrained peptide from bulk solvent to membrane surface, $\Delta G^{S}_{h\to 0}$ restraints removal for surface configuration, $\Delta G^{S\to D}_{h}$ transition of restrained peptide from surface to deep configuration, $\Delta G^{D}_{h\to 0}$ restraints removal for deep configuragion.

Results for Charmm 36 force field

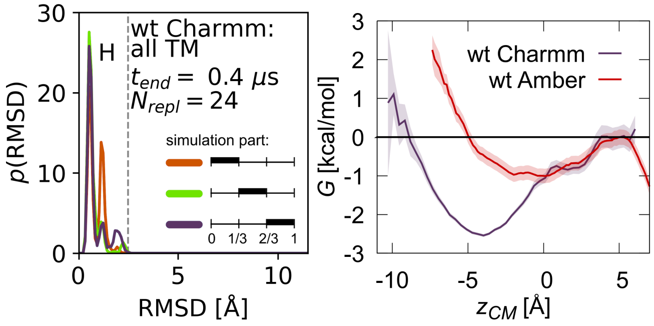

Figure 5: Left: the distribution of root mean square deviation (RMSD) of wt peptide backbone atoms with respect to tight helical hairpin (NMR structure, PDB id 2kxa, model 1) at 310 K from temperature replica exchange (tREMD) simulations conducted with Charmm 36 force field.[1] Right: the potential of mean force for wt transmembrane hairpin configuration obtained with Charmm 36 force field compared to Amber99SB-ILDNP*[2] (peptide) with Amber Lipid14[3] (lipids).

Supervised peptide insertion: N terminus

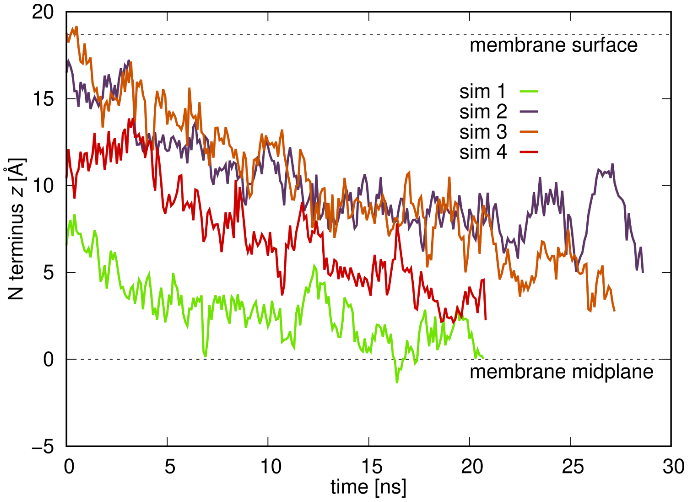

Figure 6: Four trajectories of supervised peptide insertion using the z coordinate of the N-terminal amino group as a measure of insertion progression. Trajectory #2 is based on 10 simulation rounds, trajectories #1, #3, #4 were extended to 13, 12, 11 rounds, respectively, to check that peptide does not progress further towards membrane core.

Tryptophan fluorescence quenching

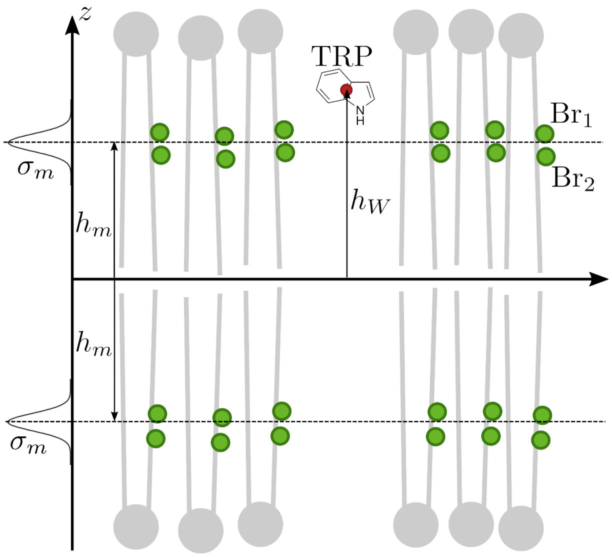

Figure 7: Spatial relations in the Ladokhin model for the estimation of depth-dependent tryptophan fluorescence quenching by brominated lipids. h_m, σ_m: average position of Br atoms with respect to membrane centre and its standard deviation, respectively, h_W: position of Trp indole ring centre with respect to membrane centre.

The ratio of tryptophan fluorescence quenching by lipids specifically brominated at position m, to fluorescence without the quencher, $\frac{F_m}{F_0}$, twas estimated using the Ladokhin model,[4] according to the following formula:

$$\frac{F_m}{F_0} = \sum_{W \in \{14,21\}} \Big\langle \exp\big(- G(h_W - h_m, \sigma_m, S) - G(h_W + h_m, \sigma_m, S)\big) \Big\rangle_{MD}. \qquad (1)$$

As illustrated in Fig. 7, h_m and σ_m are average position of Br atoms with respect to membrane centre and its standard deviation, respectively, h_W is the position of tryptophan indole ring centre along the z axis, and $G(h_W - h_m, \sigma_m, S) = \frac{S}{\sigma_m \sqrt{2\pi}} \exp\big(\frac{(h_W - h_m)^2}{\sigma_m^2}\big)$. The two Gaussian terms describe contributions from both membrane leaflets, with S being the assumed quenching intensity. The values of h_m and σ_m were calculated based on pure POPC simulations using the positions of hydrogen atoms corresponding to substituted Br atoms (Table 1). The $\frac{F_m}{F_0}$ was calculated as an ensemble average, with h_W values taken from simulation frames corresponding to the considered peptide configuration.

Table 1: Average positions and their standard deviations for Br atoms in doubly substituted brominated lipids.

Br	h_m/Å	σ_m/Å
4,5	11.6	0.5
6,7	9.9	0.6
9,10	7.5	0.7
11,12	6.3	0.7

Experimental results

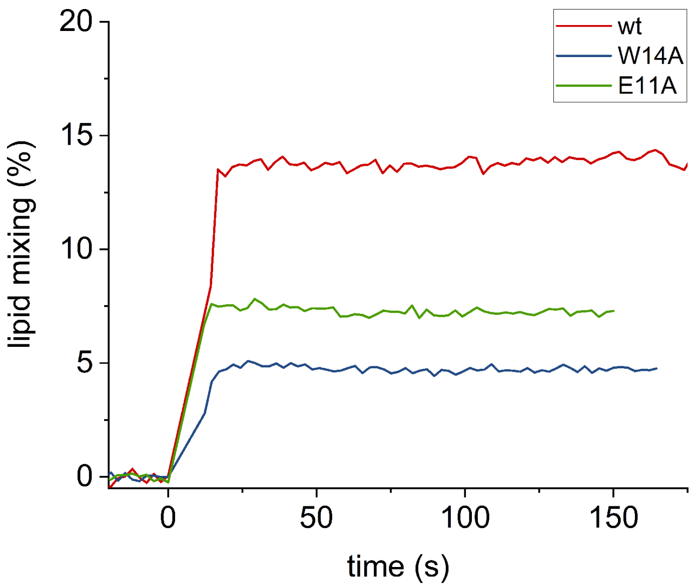

Figure 8: Averaged curves showing the percentage of lipid mixing of POPC LUVs induced by the peptides (FRET assay).

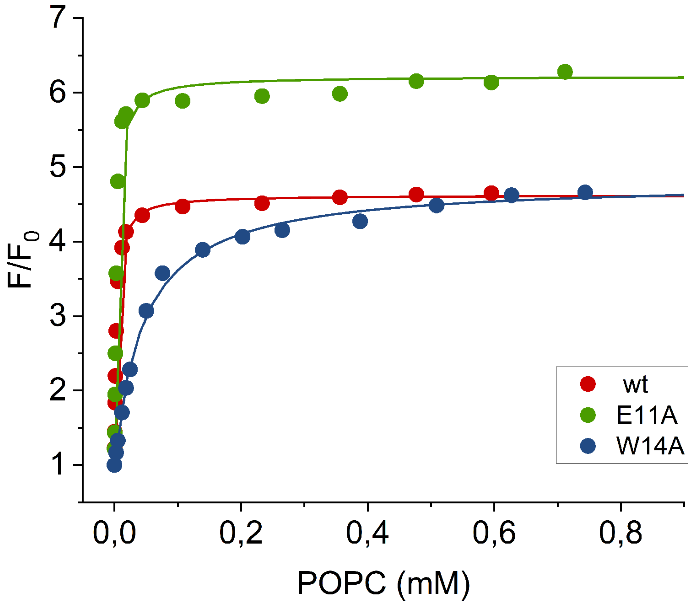

Figure 9: Sample curves showing binding of the peptides to POPC SUVs (corrected for the light scattering).

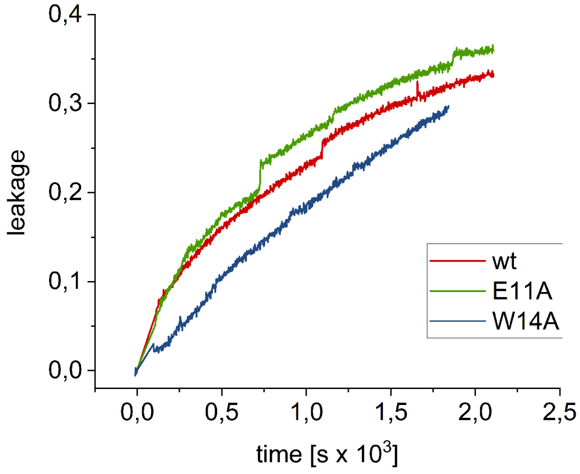

Figure 10: Sample curves of peptide-induced calcein leakage from POPC LUVs at 1:500 peptide/lipid concentration.

Simulations

Most MD runs reported in this study were based on tREMD scheme (24 or 40 replicas), with the following temperatures generated according to algorithm proposed by Patriksson and van der Spoel:[5] 310.00, 311.57, 313.15, 314.74, 316.34, 317.94, 319.54, 321.16, 322.78, 324.41, 326.05, 327.69, 329.34, 331.00, 332.66, 334.33, 336.01, 337.69, 339.39, 341.09, 342.80, 344.51, 346.23, 347.97, 349.70, 351.45, 353.20, 354.96, 356.73, 358.51, 360.29, 362.08, 363.88, 365.69, 367.50, 369.33, 371.16, 373.00, 374.84, 376.70 K.

Table 2: Summary of tREMD runs for unrestrained peptides starting at different configurations.

simulation	N replicas	total t [μs]
wt surface boomerang	24	24×1.4
wt surface hairpin	40	40×1.3
wt TM	24	24×0.3
wt$^-$ TM	24	24×0.3
wt Charmm TM	24	24×0.4
W14A surface boomerang	24	24×1.4
W14A surface hairpin	40	40×1.0
W14A TM	24	24×0.3
E11A surface boomerang	24	24×0.5
E11A surface hairpin	40	40×0.4
E11A TM	24	24×0.4

Tables 3 to 5 include information concerning the centres (z_0) of umbrella potentials used for free energy simulations, total simulation time per replica at each window t_t, simulation time actually used for PMF calculations, t_u (starting from certain point in time, till the end of run), and p values for Kolomogorof-Smirnoff test assessing the convergence of z distrbutions. All simulations were conducted as tREMD with 24 replicas.

Table 3: PMF: wt

z_0 [Å]	t_t [ns]	t_u [ns]	p
-10	50	19.8	0.101
-9	40	24.6	0.743
-8	40	25.6	0.139
-6	38	36.5	0.620
-5	35	11.1	0.162
-4	48	48.3	0.233
-3	125	50.0	0.158
-2	60	14.4	0.308
-1	105	95.7	0.336
0	60	38.1	0.347
1	60	25.1	0.226
2	90	37.8	0.281
3	110	109.5	0.330
4	111	102.2	0.465
5	140	75.3	0.390
5.5	255	51.0	0.897
6	415	215.4	0.334
7	180	50.4	0.247
8	175	45.5	0.630
9	90	32.3	0.388
10	65	41.5	0.623
11	60	16.7	0.623
12	60	29.9	0.258
13	60	55.0	0.305
14	157	56.4	0.281
15	60	47.8	0.110
16	60	56.3	0.117
18	60	47.8	0.139
20	20	8.3	0.380
22	40	18.3	0.568
24	31	27.5	0.300
26	40	31.0	0.950
27	75	28.4	0.245
28	120	28.8	0.207
29	60	35.7	0.255
30	40	40.0	0.231
32	40	28.0	0.194
33	60	60.0	0.598
34	25	23.4	0.347
36	25	24.4	0.369

Table 4: PMF: W14A

z_0 [Å]	t_t [ns]	t_u[ns]	p
-6	45	38.6	0.381
-5	45	34.9	0.233
-4	45	25.9	0.231
-3	45	17.0	0.521
-2	40	39.9	0.778
-1	60	19.1	0.266
0	80	77.6	0.373
1	60	54.8	0.491
2	60	39.5	0.270
3	60	42.9	0.301
4	90	89.8	0.203
5	210	129.8	0.279
5.5	100	57.7	0.219
6	785	407.9	0.225
7	195	128.1	0.220
8	150	56.8	0.203
9	100	81.7	0.234
10	65	28.4	0.243
11	60	53.7	0.205
12	60	57.0	0.225
13	80	73.5	0.228
14	60	52.5	0.262
15	60	47.8	0.368
16	70	57.2	0.522
18	91	23.5	0.237
20	70	44.6	0.286
22	40	38.3	0.257
24	130	57.1	0.284
26	74	34.0	0.641
27	100	53.8	0.527
28	132	42.2	0.218
29	100	20.0	0.632
30	30	19.7	0.354
32	30	11.4	0.399
34	40	34.3	0.546
36	40	39.9	0.536

Table 5: PMF: E11A

z_0 [Å]	t_t [ns]	t_u[ns]	p
1	105	62.8	0.354
2	128	127.7	0.377
3	78	29.6	0.323
4	78	77.1	0.293
5	78	27.8	0.494
6	173	165.4	0.202
6.5	170	58.4	0.116
7	540	269.6	0.369
8	365	196.9	0.408
9	180	79.1	0.835
10	80	58.9	0.479
11	80	66.4	0.311
12	80	77.9	0.226
13	121	101.0	0.365
14	95	85.5	0.236
15	95	56.8	0.938
16	10	4.6	0.322
18	55	54.6	0.422
20	53	11.7	0.211
22	30	25.1	0.205

References

[1] Huang, J.; MacKerell, A. D. *J. Comput. Chem.* **2013**, *34*, 2135–2145.

[2] Lindorff-Larsen, K.; Piana, S.; Palmo, K.; Maragakis, P.; Klepeis, J. L.; Dror, R. O.; Shaw, D. E. *Proteins Struct. Funct. Bioinforma.* **2010**, *78*, 1950–1958.

[3] Dickson, C. J.; Madej, B. D.; Skjevik, Å. a.; Betz, R. M.; Teigen, K.; Gould, I. R.; Walker, R. C. *J. Chem. Theory Comput.* **2014**, *10*, 865–879.

[4] Ladokhin, A. S. *Methods Enzymol.* **1997**, *278*, 462–473.

[5] Patriksson, A.; van der Spoel, D. *Phys. Chem. Chem. Phys.* **2008**, *10*, 2073.
